# Tibiotalocalcaneal arthrodesis with headless compression screws

**DOI:** 10.1186/s13018-016-0425-7

**Published:** 2016-08-19

**Authors:** Ji-Cheng Gong, Bing-Hua Zhou, Xu Tao, Cheng-Song Yuan, Kang-Lai Tang

**Affiliations:** Department of Orthopedic Surgery, Sports Medicine Center, Southwest Hospital, Third Military Medical University, No. 30, Gaotanyan Road, Chongqing, 400038 China

**Keywords:** Tibiotalocalcaneal, Arthrodesis, Ankle, Subtalar, Fusion

## Abstract

**Background:**

Tibiotalocalcaneal arthrodesis with headless compression screws has not been previously reported. We hypothesized that these screws could be suitable for tibiotalocalcaneal arthrodesis because of their special design. This study aimed to evaluate the clinical outcomes of patients undergoing tibiotalocalcaneal arthrodesis with headless compression screws for the treatment of severe arthropathy of the ankle and subtalar joint.

**Methods:**

From 2010 to 2015, 23 patients with severe ankle and subtalar arthropathy underwent tibiotalocalcaneal arthrodesis. All surgeries were completed by a senior surgeon in the same hospital. These patients were 18~76 years (mean 54.6 years) old; the duration of their disease was 9~38 months (mean 13.2 months). The study population included 12 males and 11 females; 12 patients underwent surgery on the left and 11 on the right. Indications for surgery included avascular necrosis of the talus (*n* = 14), severe posttraumatic arthritis (*n* = 4), osteoarthritis (*n* = 2), terminal tuberculous arthritis (*n* = 1), rheumatoid arthritis (*n* = 1) and Charcot neuroarthropathy (*n* = 1). A lateral oblique incision was performed to expose the subtalar joint, and an anteromedial longitudinal incision was used to expose the ankle joint. After the articular surfaces were removed, the tibia, talus and calcaneus were carefully aligned and fixed with two headless compression screws. Patients were followed up at 6 weeks and 3, 6 and 9 months after surgery; they were evaluated by Roles and Maudsley patient satisfaction scores, the American Orthopaedic Foot & Ankle Society (AOFAS) Ankle-Hindfoot Score, visual analogue scale (VAS) score and radiographic evaluation.

**Results:**

Seventeen patients were studied, with a mean follow-up time of 6.5 months (range 5–24). The mean Roles and Maudsley patient satisfaction score was 1.41 at the last follow-up; most of the patients were satisfied with the surgery results. The mean preoperative AOFAS Ankle-Hindfoot Score was 29.6 (range 18–37), while the mean last follow-up AOFAS Ankle-Hindfoot Score was 68.5 (range 61–80). The VAS score for preoperative functional pain was 6.95 (range 3–10) compared to 1.56 (range 0–3) postoperatively (*P* < 0.001). The mean surgical duration was 57 (range 42–125) min. The mean time to union was 3.8 months (range 3–12 months); fusion of the ankle and subtalar joint was successful in all patients. One patient experienced delayed wound healing.

**Conclusions:**

Tibiotalocalcaneal arthrodesis with headless compression screws for the treatment of severe arthropathy of the ankle and subtalar joint is an effective treatment that is minimally invasive and is associated with a short operation time, high fusion rate, low incidence of complications and good postoperative recovery.

## Background

Severe ankle and subtalar arthritis is the end stage of articular cartilage damage and malformation caused by many factors, such as trauma, failure of ankle arthrodesis, talar ischaemia and necrosis, adult-acquired flat foot, severe osteoarthritis, severe rheumatoid arthritis, infection, failure of ankle replacement, severe talipes equinovarus deformity, other congenital deformities, Charcot’s disease and neuromuscular disease. Patients with severe ankle and subtalar arthritis often suffer pain or have difficulty walking, which seriously impacts their daily life [1, 2]. When conservative treatment becomes ineffective, surgery is the final treatment option.

Tibiotalocalcaneal arthrodesis is a surgical procedure that aims to alleviate pain, restore the walking gait and improve the patient’s quality of life. Fusion of both the ankle and subtalar joints is performed at the same time to maintain the stability of the hindfoot and to correct the hindfoot alignment [3, 4]. The overriding indication for tibiotalocalcaneal arthrodesis is ankle and subtalar arthritis caused by severe osteoarthritis, talar ischaemia and necrosis, or failure of ankle replacement. Early in 1906, Lexer performed tibiotalocalcaneal arthrodesis with bone allograft [5]. Subsequently, with continuous technological improvements, fixation using screws, external fixators, intramedullary nails and plates have further alleviated the pain and improved the function of patients [6–10]. Unfortunately, the screws often cannot provide enough stability and are easily broken and become loosened, and malunion and nonunion rates are relatively high. The stability of the external fixator is insufficient, and the fixator is difficult to be cared for, with high incidence rates of infection and nonunion. Plate fixation can increase the risk of nonunion for wide incision and dissection of soft tissues. Currently, more surgeons prefer using the intramedullary nail, which can provide good structural stability, axial compression and anti-rotation properties [3, 11]. However, this fixation method has a high degree of technical difficulty in inserting the nail and a relatively high rate of complications such as neurovascular injury, soft tissue irritation, deep infection and bone fracture surrounding the end of the nail in the distal tibia [3, 12]. To our knowledge, a simple tibiotalocalcaneal arthrodesis fixation method with a high union rate and low complication rate has not yet been reported.

The second generation of headless compression screws was designed using the principles of strong cannulated compression of cancellous bone and focusing on increased compressive strength and versatility. There are several types of second-generation headless compression screws. Of these, the Acutrak screw (Acumed Ltd., Hillsboro, OR, USA) used in the study is a 120-mm, headless, fully threaded, tapered, cannulated and self-tapping device marketed in 1994; it was designed to provide compression across the fracture or joint space and improve the load to failure. Because the screw is completely threaded, there is a greater surface area for fixation between the bone and the screw [13, 14]. Because the calcaneus, talar and distal tibia are mainly composed of cancellous bone, the Acutrak screw could be suitable for tibiotalocalcaneal arthrodesis.

Therefore, we attempted to perform tibiotalocalcaneal arthrodesis with headless compression screws. This study aimed to evaluate the clinical outcome of tibiotalocalcaneal arthrodesis with headless compression screws for treating severe advanced ankle and subtalar arthritis.

## Methods

### Clinical data

During 2010–2015, 23 patients with severe ankle and subtalar arthritis underwent tibiotalocalcaneal arthrodesis. All surgeries were completed by a senior surgeon in the same hospital. These patients were 18~76 years (mean 54.6 years) old, and the duration of disease was 9~38 months (mean 13.2 months). The patients included 12 males and 11 females; 12 had surgery on the left side and 11 on the right. Fourteen patients suffered talar necrosis with severe arthritis, four had severe traumatic arthritis, one had advanced tuberculous arthritis, one had rheumatoid arthritis, one had osteoarthritis, one had Charcot’s disease and one had tumour (see Table 1). All patients had a history of chronic peri-ankle pain and limited ankle movement. The indications for surgery included severe advanced ankle and subtalar arthritis caused by various factors, manifested as peri-joint pain that worsened when walking and thus seriously influenced the daily life, with ineffectiveness of 6+ months of standard conservative treatment. The contraindications to surgery included active infection, severe osteoporosis, juveniles or patients who could not tolerate surgery and asymptomatic arthritis.

**Table 1:** Diseases for tibiotalocalcaneal arthrodesis

Disease	Number of patients	Percentage (%)
Talar necrosis with severe arthritis	14	60.9
Severe traumatic arthritis	4	17.4
Advanced tuberculous arthritis	1	4.3
Rheumatoid arthritis	1	4.3
Osteoarthritis	1	4.3
Charcot’s disease	1	4.3
Tumour	1	4.3

In the patients studied, one ultimately underwent amputation due to discovery of a foot metastasis of malignant tumour 1 month after tibiotalocalcaneal arthrodesis; five others were excluded because of the use of cannulated-head compression screws. The 17 patients eventually included into the study all provided their informed consent at enrolment and the use of patients’ data for research. This study was approved by the Ethics Committee of the Southwest Hospital affiliated with the Third Military Medical University.

### Clinical evaluation and statistical methods

The clinical evaluation included three parts: subjective evaluation, clinical efficacy evaluation and imaging evaluation.

#### Subjective evaluation

Roles and Maudsley patient satisfaction scores [15, 16] were used to assess patient satisfaction as follows: 1, indicates excellent result, no symptoms; 2, indicates good result, significant improvement; 3, indicates fair result, somewhat improved; 4, indicates poor results with same or worse symptoms.

#### Clinical efficacy evaluation

The American Orthopaedic Foot & Ankle Society (AOFAS) Ankle-Hindfoot Score and visual analogue scale (VAS) pain score were used.

#### Imaging evaluation

1. Radiological joint union: Radiological union was determined when the radiography confirmed that bone trabeculae passed through the joint line or if the joint line was vague or disappeared.
2. Hindfoot alignment: Alignment was observed with the tibiocalcaneal angle and hindfoot valgus angle [17].
3. Union time: There were two criteria for union: (1) the patient had no pain symptoms at a follow-up visit and (2) there was radiological support evidence [15]. The union time was defined as the time span from the operation to the last visit.

#### Other indicators

Other indicators are the incidence rate of complications and operative time.

SPSS13.0 software (version 13.0; SPSS Inc., Chicago, IL, USA) was used for data statistics. The mean was compared with *t* test. *P* < 0.05 suggested that a difference was statistically significant.

### Surgical technique

**Fig. 1:**
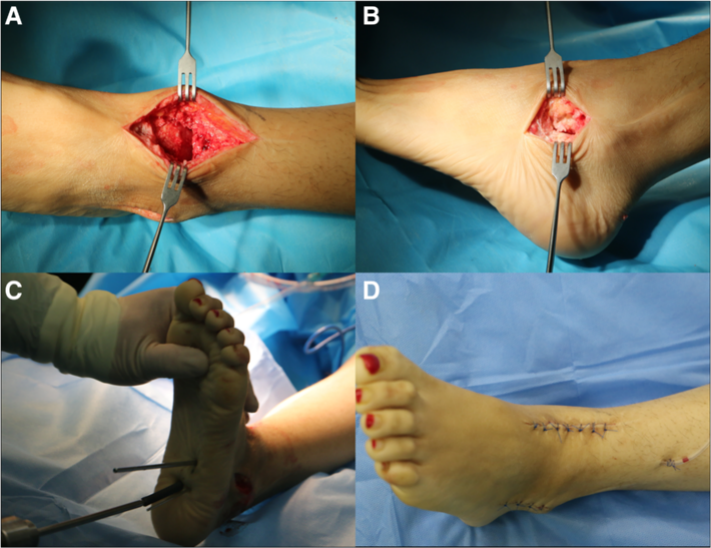
An anteromedial longitudinal incision (**a**) and lateral oblique incision (**b**) were performed, and the articular cartilage was debrided completely. The screws were twisted in along the guide wire (**c**), and then, the wound was sutured (**d**)

**Fig. 2:**
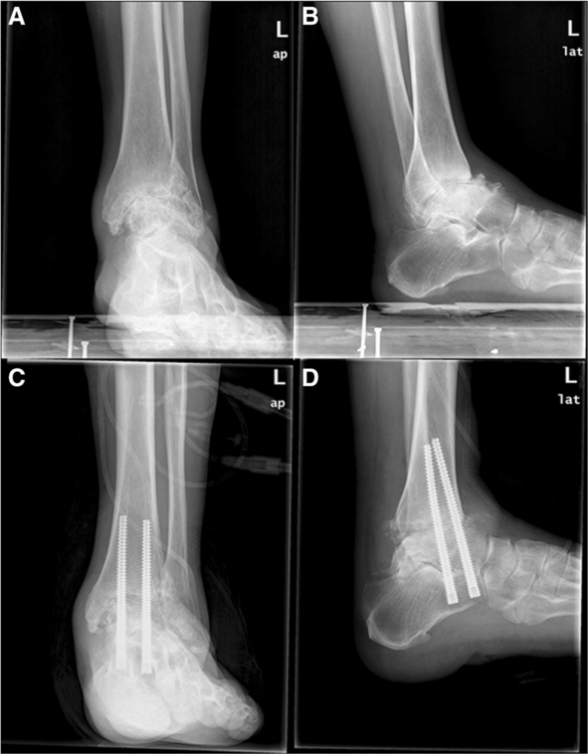
Preoperative (**a**, **b**) and postoperative (**c**, **d**) radiographs after tibiotalocalcaneal arthrodesis with headless compression screws

The patients were in a supine position with a bolster under the affected hip to facilitate internal rotation of the extremity. A pneumatic tourniquet was routinely utilized with a pressure of 300 mmHg. A 6-cm anteromedial longitudinal incision was made in the front of the ankle joint. The dissection was performed between the tibialis anterior tendon medially and the extensor hallucis longus tendon and the neurovascular bundle laterally and deepened to the periosteum to protect the adjacent soft tissues and expose the ankle joint; a 4-cm lateral oblique incision from the tip of the lateral malleolus to the basilar part of the fourth metatarsal bone crossing the tarsal sinus was performed to expose the subtalar joint (Fig. 1a, b). The cartilage was removed with an osteotome; a hole was drilled deep to the bone marrow if there was sclerosis of the subchondral bone. After the articular surface was prepared, the foot was positioned in a plantigrade position and in slight heel valgus and slight external rotation. A 4.0-mm Kirschner wire was retrogradely inserted 0.5 cm medial to the midline of the palpated lateral calcaneal pillar and 1.5 cm away from the calcaneocuboid joint. The ankle and subtalar joints were then temporarily fixed with the Kirschner wire passing through the distal end of the cortical tibial bone. Two guide wires were inserted retrogradely from the plantar surface into the distal tibia under radiographic control. It is recommended to place the wires in the posterolateral and anteromedial position of the distal tibia and calcaneus, keeping them adjacent to the medullary cavity side of the cortical bone. Before the screws were inserted, an appropriate amount of allograft bone was typically used to fill the arthrodesis site so that the bone surfaces were closely matched. Thereafter, two cannulated headless compression screws (Acumed Acutrak^②^ 120 mm) were percutaneously passed over the guide wire. We confirmed by radiography that the screws were in close contact with the medullary cavity side of the distal cortical tibial bone to achieve full axial compression. A headless compression screw was inserted in a posterior-inferior direction from the anterior distal tibia via the talus to prevent rotation as necessary. The wound was then sutured with a negative pressure drainage tube (Fig. 1cd, d; Fig. 2).

### Postoperative treatment and follow-up

After the operation, an ice compress was continued for 3–5 days. The time at which the drain was removed depended on the drainage volume; the suture was removed at 14 days. The affected limbs were protected with a foam pneumatic walker (aircast) from weight bearing for 6 weeks after surgery, and functional exercise was started once the pain was alleviated. Regular visits were made at 6 weeks, 3 months, 6 months and 1 year postoperatively. Weight bearing was allowed based on the physical examination and radiological evaluation at the follow-up visits.

## Results

All 17 patients included into the study were followed for an average of 6.5 (5–24) months. One patient experienced wound healing complications, which resolved after debridement and suturing. No complications such as poor blood supply and forefoot numbness caused by the injury of lateral plantar nerves and vessels, breakage or loosening of internal fixation or irritation of soft tissues surrounding the screws were observed.

### Subjective evaluation

**Fig. 3:**
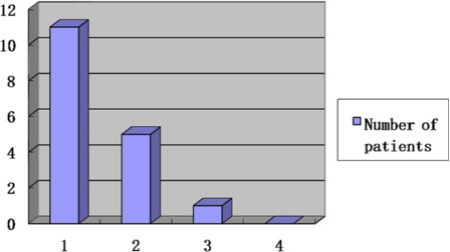
Roles and Maudsley patient satisfaction scores at the last visit

Roles and Maudsley patient satisfaction scores were 1.41 on average at the last visit, indicating that most patients experienced satisfactory functional improvement after surgery. Only one patient scored 3, meaning that their functional improvement and operation outcomes were moderate (Fig. 3).

### Clinical efficacy evaluation

The patients’ VAS pain scores decreased from 6.95 (3–10) preoperatively to 1.56 (0–6) at the time of the latest follow-up. At the last visit, the AOFAS Ankle-Hindfoot Score was 68.5/100 (61–80), which was significantly higher than 29.6/100 (18–37) before surgery. The differences were all statistically significant (Table 2).

**Table 2:** AOFAS Ankle-Hindfoot Scores and VAS pain scores before operation and at the last visit (*n* = 17, *x* ± *s*)

Score	Before operation	At the last visit	*t* value	*P* value
AOFAS score	29.6 ± 5.3	68.5 ± 3.4	13.09	0.0000
VAS pain score	6.95 ± 0.3	1.56 ± 0.6	20.84	0.0000

### Imaging evaluation

All patients achieved bony fusion at a mean union time of 3.8 (3–12) months. The mean hindfoot extroversion angle was 3.5° (from 3° introversion to 6° extroversion). The tibiocalcaneal angle was 93.5° (85°–105°) (Fig. 2).

### Other indicators

The operation time was 57 (42–125) min. Only one patient experienced poor surgical wound healing; the incidence rate of complications was 5.9 %. This patient was rehospitalized for debridement and suturing, and thereafter, the wound healing was good. No other patients required additional surgery.

## Discussion

There are many forms of fixation for tibiotalocalcaneal arthrodesis, including screws, plates, intramedullary nails and external fixators. The development of the second-generation headless compression screw is an innovation that allows surgeons to perform arthrodesis. In the present study, the outcomes of the patients who underwent tibiotalocalcaneal arthrodesis using headless compression screws are encouraging. As the previous reports about tibiotalocalcaneal arthrodesis with intramedullary nails confirmed, the AOFAS Ankle-Hindfoot Score was 63–71, the satisfaction rate was 78–92 % [18], VAS pain score was 1.98 on average and Roles and Maudsley patient satisfaction scores were 1.77 on average [15]. Our study results showed that after surgery, the AOFAS Ankle-Hindfoot Score was 68.5 on average, the satisfaction rate was 94.1 % and VAS pain score was 1.56 on average; Roles and Maudsley patient satisfaction scores at the last visit were 1.41 on average. Nearly all patients who underwent tibiotalocalcaneal arthrodesis with headless compression screws experienced satisfactory postoperative outcomes. Jehan et al. [19] systematically reviewed 33 studies and analysed the efficacy of tibiotalocalcaneal arthrodesis with 659 intramedullary nails in 631 patients. They found that the union rate was 86.7 %, the average union time was 4.5 months and the incidence of complications was 55.7 %. Fang et al. [18] reported that the operation time was 128 (72–214) min. The present study showed that the hindfoot alignment was effectively corrected for all patients, the average union time is 3.8 months, the union rate was 100 %, the incidence rate of complications was 5.9 % and the operation time was 57 (42–125) min. Compared with tibiotalocalcaneal arthrodesis with the intramedullary nail, the technique using a headless compression screw had a shorter operation time, a significantly lower incidence rate of complications, a higher union rate and a shorter fusion time.

Several factors are responsible for these advantages. First, the posterior tibial artery and the tibial nerve split into two branches just posterior to the medial malleolus; the lateral neurovascular branch originates at the medial malleolus and passes longitudinally, crossing the long flexor tendons and flexor accessorius to lie close to the base of the fifth metatarsal; and the medial neurovascular branch originates at the medial malleolus and reaches the first metatarsal base [20]. In present study, the guide wire was placed in the posterolateral and anteromedial position of the heel pad percutaneously; meanwhile, compared with the tibiotalocalcaneal arthrodesis using an intramedullary nail, gradient reaming and the use of proximal and distal locking bolts were not required in the tibiotalocalcaneal arthrodesis with headless compression screws, such that it is likely to reduce the risks of nerve and vascular injury and decrease operative time.

Second, although the calcaneus, talar and distal tibia are mainly composed of the cancellous bone, the cortical bone (especially the cortical bone of the medial slope of the anterior portion of the calcaneus) and the bone adjacent to its medullary cavity side were relatively dense, so the headless compression screw which was placed in this position could produce a better grasping force and subsequently a better axial compression force; to make full use of the good grasping force and powerful axial compression of the screws, we designed a screw insertion procedure specific to the anatomic characteristics of the tibiotalocalcaneal joint. In the calcaneus side, the posterolateral headless compression screw was placed in close contact with the posterolateral medullary cavity side of the cortical bone as in the distal tibial, and the entry point of the anteromedial screw was on the medial slope of the anterior portion of the calcaneus. Besides, the screws were parallel to each other, so they were quite likely to provide a rigid fixation and a strong axial compression across the arthrodesis site [13, 14]. Placing screws anterograde into the ankle and retrograde in the subtalar joint is a traditional fixation method; however, at least four screws are used to achieve a fixation across the arthrodesis site and it is difficult to keep the screws parallel to provide a strong axial compression, so we hypothesized that the expected stability of the fixation method should be inferior to our method. Furthermore, the distal tibia was approximately a tetragon, and the screw insertion on its diagonals could provide enough space to facilitate the insertion of more screws.

Third, the appropriate and minimally invasive incisions helped to protect the blood supply of the arthrodesis site and reduce the surgical time. A longitudinal incision at the anterolateral ankle along the anterior fibula is commonly used for intramedullary nail fixation, while a lateral malleolus osteotomy may be performed occasionally to provide good exposure. However, such an incision can damage the local blood supply, injure the nerves and vessels and result in a high infection rate. We have confirmed that a lateral oblique incision from the tip of the lateral malleolus to the basilar part of the fourth metatarsal bone could provide good exposure of the subtalar joint in a relatively short time [21]. Meanwhile, an anteromedial longitudinal incision can provide good exposure of the ankle joint. Furthermore, these two incisions could reduce damage to adjacent soft tissues, avoid contacting the nerve and provide good exposure, which is beneficial to the correction of hindfoot deformity and removal of articular cartilage. In addition, these incisions were nearly parallel and their distance was >5 cm, so the skin between them was not at risk for necrosis. In this study, only one patient experienced poor wound healing at the anterior ankle, which resulted from an inflammatory reaction to the allograft bone and inadequate drainage after the premature removal of the drainage tube, leading to increased effusion. After debridement and suturing, the wound healed well. Based on the aforementioned advantages, we recommend the combined incision for tibiotalocalcaneal arthrodesis with headless compression screws.

This study has some shortcomings. First, it was not a randomized, double-blinded, controlled clinical study, and the level of evidence was insufficient. Second, because the tibiotalocalcaneal arthrodesis is an end-stage procedure, it is uncommon in our centre. Furthermore, to get a better result, we only included patients using a uniform type of screws in the case series, five patients were excluded because of the use of cannulated-head compression screws, so the sample size was relatively small. This study aimed to evaluate the early outcomes of tibiotalocalcaneal arthrodesis with headless compression screws, and the last follow-up was defined at the time when the patients achieved bony fusion, so the average follow-up was low. But whether the procedure could lead to a premature deterioration of the neighbouring articulations should be detected with longer follow-up, so we will increase the number of cases and examine the outcome with longer follow-up in the future study. Third, future biomechanical research is necessary to determine the construct stability of various fixation methods.

## Conclusions

The preliminary clinical results have confirmed that tibiotalocalcaneal arthrodesis with headless compression screws is an effective fixation technique for treating severe ankle and subtalar lesions; it has many advantages including being minimally invasive, with a short operation time, a high fusion rate, a low rate of complications and a good postoperative recovery.

## Abbreviations

VAS: Visual analogue scale
AOFAS: American Orthopaedic Foot & Ankle Society

## References

[1] Franceschi F, Franceschetti E, Torre G, Papalia R, Samuelsson K (2016). Tibiotalocalcaneal arthrodesis using an intramedullary nail: a systematic review. Knee Surg Sports Traumatol Arthrosc.

[2] Vilà y Rico J, Rodriguez-Martin J, Parra-Sanchez G, Marti Lopez-Amor C (2013). Arthroscopic tibiotalocalcaneal arthrodesis with locked retrograde compression nail. J Foot Ankle Surg.

[3] Shah KS, Younger AS (2011). Primary tibiotalocalcaneal arthrodesis. Foot Ankle Clin.

[4] Burks JB (2012). Tibiotalocalcaneal arthrodesis. Clin Podiatr Med Surg.

[5] Mendicino RW, Catanzariti AR, Saltrick KR, Dombek MF, Tullis BL (2004). Tibiotalocalcaneal arthrodesis with retrograde intramedullary nailing. J Foot Ankle Surg.

[6] Tsailas PG, Wiedel JD (2010). Arthrodesis of the ankle and subtalar joints in patients with haemophilic arthropathy. Haemophilia.

[7] Krissen C, Sumon H, Nicholas B, Howard C, Andrew A (2011). Tibio-talo-calcaneo fusion using a locked intramedullary compressive nail. Foot Ankle Surg.

[8] DiDomenico LA, Wargo-Dorsey M (2012). Tibiotalocalcaneal arthrodesis using a femoral locking plate. J Foot Ankle Surg.

[9] Jeong ST, Park HB, Hwang SC, Kim DH, Nam DC (2012). Use of intramedullary nonvascularized fibular graft with external fixation for revisional Charcot ankle fusion: a case report. J Foot Ankle Surg.

[10] Nielsen KK, Linde F, Jensen NC (2008). The outcome of arthroscopic and open surgery ankle arthrodesis: a comparative retrospective study on 107 patients. Foot Ankle Surg.

[11] Thomas RL, Sathe V, Habib SI (2012). The use of intramedullary nails in tibiotalocalcaneal arthrodesis. J Am Acad Orthop Surg.

[12] Fenton P, Bali N, Matheshwari R, Youssef B, Meda K (2014). Complications of tibio-talar-calcaneal fusion using intramedullary nails. Foot Ankle Surg.

[13] Toby EB, Butler TE, McCormack TJ, Jayaraman G (1997). A comparison of fixation screws for the scaphoid during application of cyclical bending loads. J Bone Joint Surg Am.

[14] Fowler JR, Ilyas AM (2010). Headless compression screw fixation of scaphoid fractures. Hand Clin.

[15] Thomas AE, Guyver PM, Taylor JM, Czipri M, Talbot NJ (2015). Tibiotalocalcaneal arthrodesis with a compressive retrograde nail: a retrospective study of 59 nails. Foot Ankle Surg.

[16] Roles NC, Maudsley RH (1972). Radial tunnel syndrome: resistant tennis elbow as a nerve entrapment. J Bone Joint Surg Br.

[17] Gross JB, Belleville R, Nespola A, Poircuitte JM, Coudane H (2014). Influencing factors of functional result and bone union in tibiotalocalcaneal arthrodesis with intramedullary locking nail: a retrospective series of 30 cases. Eur J Orthop Surg Traumatol.

[18] Fang Z, Claaßen L, Windhagen H, Daniilidis K, Stukenborg-Colsman C (2015). Tibiotalocalcaneal arthrodesis using a retrograde intramedullary nail with a valgus curve. Orthop Surg..

[19] Jehan S, Shakeel M, Bing AJ, Hill SO (2011). The success of tibiocalcaneal arthrodesis with intramedullary nailing—a systematic review of the literature. Acta Orthop Belg.

[20] Knight T, Rosenfeld P, Jones IT, Clark C, Savva N (2014). Anatomic structures at risk: curved hindfoot arthrodesis nail—a cadaveric approach. J Foot Ankle Surg.

[21] Yuan CS, Tan XK, Zhou BH, Liu JP, Tao X (2014). Differential efficacy of subtalar fusion with three operative approaches. J Orthop Surg Res..

